# Supplementary material for: Technology-assisted platform (TAP) for training and supervision of task-shared psychosocial interventions to ensure competency during scale-up in low-resource settings
Source: Glob Ment Health (Camb). 2026 Apr 22;13:e92. doi: 10.1017/gmh.2026.10202 (PMC13161798; doi:10.1017/gmh.2026.10202)
Supplement: Atif et al. supplementary material [file S2054425126102027sup001.docx]

| **Supplement file 1: Competency Assessment Scores – Peers** | | | | | | | | | | | | |
| --- | --- | --- | --- | --- | --- | --- | --- | --- | --- | --- | --- | --- |
| **ENACT SCORES** | | | | | | | | | | | | |
| **Items** | **Post Training** | | | | **6 months post training** | | | | **12 months post training** | | | |
|  | Level  1 | level  2 | level  3 | level  4 | Level 1 | Level  2 | Level  3 | Level  4 | Level 1 | Level  2 | Level  3 | Level  4 |
| Non-verbal communication | 0% | 84% | 12% | 4% | 0% | 58% | 32% | 12% | 0% | 5% | 48% | 48% |
| Verbal communication | 0% | 88% | 10% | 2% | 0 % | 66% | 34% | 2% | 0% | 7% | 55% | 39% |
| Explain and promote confidentiality | 0% | 92% | 6% | 2% | 0% | 76% | 24% | 2% | 0% | 14% | 68% | 18% |
| Rapport building and self-disclosure | 0% | 96% | 4% | 0% | 0% | 86% | 16% | 0% | 0% | 0% | 77% | 23% |
| Exploration and normalisation of feelings | 0% | 100% | 0% | 0% | 0% | 88% | 14% | 0% | 0% | 11% | 75% | 14% |
| Demonstrate empathy warmth, & geniuses | 0% | 96% | 4% | 0% | 0% | 68% | 32% | 2% | 0% | 5% | 68% | 27% |
| Assessment of harm, & developing response plan | 0% | 100% | 0% | 0% | 0% | 98% | 4% | 0% | 0% | 48% | 48% | 5% |
| Connect to social functioning and impact on life | 0% | 96% | 4% | 0% | 0% | 92% | 10% | 0% | 0% | 20% | 75% | 5% |
| Explore client's explanation for problem | 0% | 100% | 0% | 0% | 0% | 92% | 10% | 0% | 0% | 30% | 66% | 5% |
| Involvement of family and significant others | 0% | 96% | 4% | 0% | 0% | 62% | 38% | 2% | 0% | 7% | 86% | 7% |
| Collaborative goal setting | 0% | 86% | 14% | 0% | 0% | 88% | 14 | 0% | 0% | 23% | 68% | 9% |
| Promote realistic hope for change | 0% | 90% | 10% | 0% | 0% | 76% | 26% | 0% | 0% | 9% | 81% | 9% |
| Incorporate coping mechanism and prior solution | 0% | 98% | 2% | 0% | 0% | 92% | 8% | 2% | 0% | 34% | 59% | 7% |
| Psychoeducation with local terminology | 0% | 90% | 10% | 0% | 0% | 30% | 72% | 0% | 0% | 0% | 48% | 52% |
| Elicitation of feedback | 0% | 100% | 0% | 0% | 0% | 92% | 10% | 0% | 0% | 25% | 68% | 7% |
| **Thinking Healthy Competency Scores** | | | | | | | | | | | | |
| **Items** | **Post Training** | | | | **6 months post training** | | | | **12 months post training** | | | |
|  | **Level**  **1** | **level**  **2** | **level**  **3** | **level**  **4** | **Level 1** | **Level**  **2** | **Level**  **3** | **Level**  **4** | **Level 1** | **Level**  **2** | **Level**  **3** | **Level**  **4** |
| Mood & activity monitoring | 0% | 88% | 0% | 0% | 0% | 58% | 44% | 0% | 0% | 34% | 64% | 2% |
| Psychoeducation about thoughts, feelings, & behaviours | 0% | 62% | 38% | 0% | 0% | 16% | 80% | 6% | 0% | 0% | 75% | 25% |
| Linking thoughts, feelings & behaviours: connecting thoughts & feelings with personal experience | 0% | 94% | 6% | 0% | 0% | 38% | 64% | 0% | 0% | 9% | 73% | 18% |
| Linking thoughts, feelings & behaviours: connecting feelings with behaviours | 0% | 84% | 16% | 0% | 0% | 26% | 76% | 0% | 0% | 2% | 81% | 16% |
| Identifying more difficult & unhelpful thoughts | 0% | 92% | 8% | 0% | 0% | 54% | 46% | 2% | 0% | 18% | 68% | 14% |
| Developing new thoughts, feeling, behaviours & associations: creating alternative thoughts | 0% | 92% | 8% | 0% | 0% | 70% | 32% | 0% | 0% | 20% | 68% | 11% |
| Developing new thoughts, feeling, behaviours & associations: differences between new & previous thoughts | 0% | 98% | 2% | 0% | 0% | 66% | 36% | 0% | 0% | 23% | 61% | 16% |
| Using thought records with in-session practice | 0% | 90% | 10% | 0% | 0% | 62 | 38% | 2% | 0% | 23% | 70% | 7% |
| Reviewing thought records/homework | 0% | 86% | 14% | 0% | 0% | 64% | 36% | 2% | 0% | 27% | 57% | 16% |
| Using a role-play to build communication skills & improve relationships | 0% | 100% | 0% | 0% | 0% | 80% | 22% | 0% | 0% | 86% | 14% | 0% |
| Stress management: introducing a new strategy (then practice & repeat) | 0% | 90% | 10% | 0% | 0% | 66% | 32% | 4% | 0% | 27% | 36% | 36% |
